# Efficacy of 3D Culture Priming is Maintained in Human Mesenchymal Stem Cells after Extensive Expansion of the Cells

**DOI:** 10.3390/cells8091031

**Published:** 2019-09-05

**Authors:** Thomas J. Bartosh, Joni H. Ylostalo

**Affiliations:** 1 Internal Medicine, Texas A&M University Health Science Center, Temple, TX 76508, USA; 2 Department of Biology, University of Mary Hardin-Baylor, Belton, TX 76513, USA

**Keywords:** MSC, spheroid, 3D, anti-inflammatory, immunomodulatory, passage, culture-expansion, xeno-free, TSG-6, PGE2

## Abstract

The use of non-optimal preparations of mesenchymal stem cells (MSCs), such as extensively expanded cells, might be necessary to obtain the large numbers of cells needed for many clinical applications. We previously demonstrated that minimally expanded (early passage) MSCs can be pre-activated as spheroids to produce potentially therapeutic factors in 3D cultures. Here, we used extensively expanded (late passage) MSCs and studied their 3D-culture activation potential. MSCs were culture-expanded as 2D monolayers, and cells from various passages were activated by 3D culture in hanging drops with either fetal bovine serum (FBS)-containing media or a more clinically-applicable animal product-free (xeno-free) media. Gene expression analyses demonstrated that MSC spheroids prepared from passage 3, 5, and 7 cells were similar to each other but different from 2D MSCs. Furthermore, the expression of notable anti-inflammatory/immune-modulatory factors cyclooxygenase-2 (PTGS2), TNF alpha induced protein 6 (TNFAIP6), and stanniocalcin 1 (STC-1) were up-regulated in all spheroid preparations. This was confirmed by the detection of secreted prostaglandin E2 (PGE-2), tumor necrosis factor-stimulated gene 6 (TSG-6, and STC-1. This study demonstrated that extensively expanded MSCs can be activated in 3D culture through spheroid formation in both FBS-containing and xeno-free media. This work highlights the possibility of activating otherwise less useable MSC preparations through 3D culture generating large numbers of potentially therapeutic MSCs.

## 1. Introduction

The nonhematopoietic multipotent stromal cells first identified in bone marrow, commonly referred to as MSCs (mesenchymal stem cells), are poised to transform cell-based therapies due to their many beneficial properties [1]. Broadly defined, MSCs constitute a heterogenous subset of plastic-adherent spindle-shaped cells capable of differentiating, with proper stimulus, into the mesodermal cell types that form bone, cartilage, and adipose tissue [2,3,4,5]. These properties were exploited in early MSC-based applications focused on regeneration of connective tissues [5,6] and to determine the existence of similar cells throughout the body [7,8].

More recently, there has been considerable interest in harnessing the profound paracrine-mediated immunomodulatory effects of MSCs [6,9,10,11,12,13]. In response to cytokines and other biochemical signals liberated from injured tissues, MSCs, recruited to the injury site, secrete numerous factors that collectively regulate the signaling pathways and cell types associated with inflammatory responses and immunological phenotypes. These activities, in combination with their lack of class II major histocompatibility complex proteins (MHC) and costimulatory factors, potentially allow the cells to evade alloreactive immune response [9,10,11,12,13]. Accordingly, MSCs are being explored as a viable treatment option to attenuate tissue damage caused by severe inflammatory microenvironments, such as those observed with graft-vs-host-disease, autoimmune disease, ischemic tissue injury, and organ transplants [14,15].

Therapies utilizing MSCs often require ex vivo expansion to generate the large numbers of cells needed for patients and to overcome limitations in tissue recovery [16]. Ex vivo expansion has been particularly important for bone marrow MSCs, which remain the primary clinical source but only constitute about 0.01% of all mononuclear cells in the bone marrow, a fraction that declines with age [17].

Typically, MSCs have been almost exclusively expanded on rigid tissue culture-treated polystyrene as a two-dimensional (2D) monolayer [16]. These culture conditions were vital for the initial discovery of MSCs [18] and for characterizing their multipotency [3,4]. While convenient and highly effective for cell expansion, 2D cultures are highly artificial and overlook the importance of cell signaling networks activated by cell–cell and cell–matrix interactions in native tissues [19].

Expansion under 2D or similar conditions alters the differentiation potential of MSCs [20,21] and has been shown to be detrimental to the migratory and immunomodulatory capacity of the cells [22]. Extensive expansion has also been considered to cause inconsistent results in clinical studies capitalizing on the immunomodulatory potential of the cells [23,24], raising questions as to the best methods for preparing the cells prior to administration into patients [25]. With demands for MSCs increasing, finding effective and economical ways to preserve the therapeutic properties of culture-expanded cells has become critical. It has also become important to improve standardized methods for culturing the cells.

Over the past several years, we and others have studied MSCs in various 3D culture formats in an attempt to recapitulate the cell–cell and cell–matrix interactions of native tissue. Culturing the cells in 3D has been shown to enhance many of the therapeutic properties of MSCs including their immunosuppressive, immunomodulatory, and anti-cancer effects [26] We previously showed that MSCs spontaneously aggregate in 3D culture, forming spheroids primed to secrete a variety of potentially therapeutic anti-inflammatory factors, such as prostaglandin E2 (PGE2), tumor necrosis factor-stimulated gene 6 (TSG-6), and stanniocalcin 1 (STC-1) [25,26,27,28]. Importantly, the secretion of these factors by MSCs in cultured spheroids mimics the activation of MSCs that aggregate in vivo after injection into animals [29,30,31]. Moreover, the important roles for PGE2, TSG-6, and STC-1 in the regulation of the monocyte/macrophage phenotype and function is of particular interest [28,31,32,33,34,35,36,37,38,39,40].

Here, we employed a 3D culture protocol consisting of suspending minimally expanded (early passage) and extensively expanded (late passage) MSCs in hanging drops of fetal bovine serum (FBS)-containing media and xeno-free (i.e., free of animal components) media, thus resulting in activated MSCs in spheroids. Our results showed that the production of anti-inflammatory factors PGE2, TSG-6, and STC-1 can be restored in late passage MSCs in 3D cultures. These results suggest that the utilization of 3D culture techniques might circumvent issues surrounding the altered cellular properties of extensively expanded MSCs.

## 2. Materials and Methods

### 2.1. MSC Culture

Human MSCs, obtained from bone marrow of three healthy adult males (24–37 years of age) were provided as passage 1 cells by the Center for the Preparation and Distribution of Adult Stem Cells at Texas A&M University Health Science Center (https://medicine.tamhsc.edu/centers/irm/msc-distribution.html). The MSCs were isolated from a 1–4 mL bone marrow aspirate of the iliac crest. Nucleated cells were obtained by density gradient centrifugation (Ficoll-Paque; GE Healthcare; Chicago, IL, USA) and were resuspended in a complete culture medium (CCM) consisting of α-Minimum Essential Medium (MEMα, Gibco, Thermo Fisher Scientific, Waltham, MA, USA) supplemented with 17% fetal bovine serum (FBS, Atlanta Biologicals, Flowery Branch, USA), 100 units/mL penicillin (Gibco), 100 μg/mL streptomycin (Gibco), and 2 mM L glutamine (Gibco). Cells were seeded in 175 cm^2^ flasks (Nunc, Thermo Fisher Scientific), and were subsequently cultured at 37 °C in a humidified atmosphere with 5% CO_2_ for 24 h. Non-adherent cells were discarded, while adherent cells were incubated 4–11 days until approximately 70% confluent. Cells were harvested with 0.25% trypsin and 1 mM ethylenediaminetetraacetic acid (EDTA, Gibco), and they were re-plated at 50 cells/cm^2^ in an intercommunicating system of culture flasks (Nunc, Thermo Fisher Scientific). The cells were incubated for 7–12 days until approximately 70% confluent, harvested with trypsin/EDTA, and frozen as passage 1 cells in MEMα containing 30% FBS and 5% dimethyl sulfoxide (DMSO; Sigma; St. Louis, MO, USA). Prior to distribution, the passage 1 cells were characterized by the Center and reported to meet all criteria for the MSC phenotype established by the International Society for Cellular Therapy [4]. Here, frozen vials of the passage 1 MSCs from the three donors were thawed, suspended in CCM, and plated on a 152 cm^2^ culture dish (Corning). After 24 h, cells were harvested using trypsin/EDTA and plated at 100 cells/cm^2^ for 7 days, with medium changes at days 3 and 6, before freezing as passage 2 cells. For the experiments described here, a vial of passage 2 MSCs were recovered by plating the cells in CCM on a 152 cm^2^ culture dish for 24 h. Cells were harvested with trypsin/EDTA, re-seeded at 100 cells/cm^2^ in CCM, and incubated for 7 days with medium change at days 3 and 6. This was repeated until the cells did not double in their number in 7 days. Triplicate counts of the cells after each passage were obtained using a hemocytometer. Prior to use in experiments herein, MSC surface markers were corroborated by flow cytometry (Supplemental Figure S1, Supplemental Table S1). For some experiments, MSCs were cultured after passage 3, 5, 7, and 9 at a very high density of (Adh VH), 200,000 cells/cm^2^ at 714 cells/μL for 3 days to match the spheroid generation conditions.

### 2.2. Generation of Spheroids and Spheroid-Derived Cells

MSC spheroids were generated as previously described [26]. Briefly, MSCs from passages 3, 5, 7, and 9 were plated in hanging drops on an inverted culture dish lid in 35 μL of a culture medium at 25,000 cells/drop. The lid was then rapidly re-inverted onto the culture dish that contained phosphate buffered saline (PBS, Gibco) to prevent evaporation of the drops. The hanging drop cultures were incubated for 3 days at 37 °C in a humidified atmosphere with 5% CO_2_. The MSCs in hanging drops were cultured in two different media formulations, CCM (containing FBS) and a xeno-free (XF) formulation comprising a StemPro XF (Life Technologies, Thermo Fisher Scientific) base medium supplemented with clinical grade human serum albumin (HSA, 13 mg/mL) isolated from human blood (Baxter, Deerfield, IL, USA) [25]. To obtain sphere-derived cells, spheroids were collected from the tissue culture dish lid using a cell lifter (Corning, Tewksbury, MA, USA), washed in PBS, and then incubated with trypsin/EDTA at 37 °C for approximately 10 min with pipetting every 2–3 min. Spheroid-derived cells were collected by centrifugation at 453× *g* for 10 min.

### 2.3. Conditioned Media and Cell Lysate Harvest

Spheroids and conditioned media, from 3–4 separate experiments, were collected from the tissue culture dish lid using a cell lifter and centrifuged at 453× *g* for 5 min. The supernatant was clarified by centrifugation at 10,000× *g* for 10 min and stored at −80 °C. To obtain sphere cell lysates, spheres were centrifuged at 453× *g* for 5 min, washed with PBS, centrifuged at 453× *g* for 5 min, and lysed with an RLT buffer from an RNeasy Mini Kit (Qiagen, Germantown, MD, USA). For TSG-6 and STC-1 ELISA, intact spheres from 3 day hanging drop cultures were transferred to 6-well low adherent dishes (Costar, Corning) for 6 h in MEMα supplemented with 2% FBS, penicillin–streptomycin, and l-glutamine.

### 2.4. Microarrays

RNA was isolated from thawed cell lysates of P3 adherent monolayer MSCs (Adh), P3 spheroids (Sph P3), P5 spheroids (Sph P5), and P7 spheroids (Sph P7), from 3 separate experiments with the RNeasy Mini Kit. The isolated RNA was quantified with a Nanodrop spectrophotometer (Thermo Fisher Scientific), and the RNA from 3 separate biological experiments were pooled at equal amounts (100 ng each) for total of 300 ng for each sample. Labeled amplified RNA (aRNA) was prepared according to manufacturer’s instructions for the GeneChip 3′ IVT Express Kit (Affymetrix, Thermo Fisher Scientific). A total of 15 μg of labeled aRNA was fragmented and hybridized (GeneChip Hybridization Oven 640, Affymetrix) onto human arrays (HG-U133 Plus 2.0, Affymetrix), followed by washing and staining (GeneChip Fluidics Station 450, Affymetrix) with a GeneChip Wash and Stain Kit (Affymetrix). Arrays were scanned with a GeneChip Scanner (Affymetrix), and raw data files (CEL-files) were transferred into a Transcriptome Analysis Console (TAC, 4.0, Applied Biosystems, Thermo Fisher Scientific). Library files were obtained from NetAffx through the TAC software, and the data were normalized using the robust multi-chip analysis (RMA) algorithm. Principal component analysis was performed with the TAC using all the genes. For hierarchical clustering, the data were filtered using only genes that were either up- or down-regulated at least 4-fold between any of the spheroid samples and the monolayer sample, resulting in 1328 genes. To generate the Venn diagram, each spheroid sample was compared to the monolayer sample, and genes that were either up- or down-regulated at least 2-fold were used. The data were studied for pathways enriched in the differentially expressed genes between the spheroid MSCs and the adherent monolayer MSCs using the WikiPathways feature in the TAC software. The significance of a pathway was calculated using a 2 × 2 contingency in a Fisher’s exact test (two sided). The *p*-values were then converted to −log10, resulting in significance values. The data were also queried for the expression of potentially therapeutic molecules identified previously [26] and the differentially expressed interleukin 1 (IL-1) signaling molecules we identified in our previous work [27].

### 2.5. PGE2 ELISA

Conditioned medium samples (*n* = 4) were diluted to 1:50–1:100 for the determination of PGE2 concentration by the ELISA kit (R&D Systems, Minneapolis, MN, USA). Optical density was determined on a plate reader (FLUOstar Omega; BMG Labtech, Cary, NC, USA) at an absorbance of 450 nm with a wavelength correction at 540 nm to correct for the optical imperfections in the plate.

### 2.6. TSG-6/STC-1 ELISA

The level of secreted STC-1 (*n* = 4) was assessed using an ELISA kit (R&D Systems) following procedures set forth by the manufacturer. The level of TSG-6 protein secreted by MSCs (*n* = 4) was measured using an internal ELISA assay, as described previously [26,27]. Reagents for TSG-6 ELISA were purchased from R&D Systems unless otherwise indicated. Briefly, the wells of a 96-well high-binding polystyrene plate were incubated overnight with 10 µg/mL of a TSG-6 monoclonal antibody (clone A38.1.20; Santa Cruz Biotechnology, Dallas, TX, USA) diluted in PBS. Afterward, each well was washed 3–4 times with 400 µL of a 1× wash buffer and then blocked in 200 µL of a PBS solution containing 0.5% bovine serum albumin (BSA) for one hour. A conditioned medium and TSG-6 protein standards diluted in blocking buffer were applied to the appropriate wells and incubated at room temperature (RT) for 2–3 h on a plate shaker (VWR International, Radnor, PA, USA). After repeating the washes, biotinylated anti-human TSG-6 (0.5 µg/mL) in 100 µL of a blocking buffer was applied to the wells and incubated on a plate shaker for 1–2 h. The wells were washed again followed by 20 min incubation with streptavidin–horseradish peroxidase. TSG-6 and STC-1 proteins were visualized using substrate solution containing stabilized hydrogen peroxide and tetramethylbenzidine. The colorimetric reaction was terminated with 2N sulfuric acid. For all assays, optical density was measured on a plate reader (FLUOstar OMEGA, BMG Labtech) at an absorbance of 450 nm with optical imperfections corrected using a wavelength of 540 nm.

### 2.7. Macrophage Inflammatory Assay

The effect of the conditioned medium on inflammatory response was determined by the measuring of selected cytokines produced by macrophages in response to lipopolysaccharide (LPS, Sigma) stimulation, as described previously [26,28,30,41]. J774A.1 mouse monocytes/macrophages (ATCC, Manassas, VA, USA) were expanded on 15 cm petri dishes as loosely adherent cultures in high-glucose Dulbecco’s Modified Eagle Medium (DMEM, Gibco) supplemented with 10% FBS, 100 units/mL penicillin, and 100 µg/mL streptomycin. At approximately 80–90% confluency, the cells were collected and stimulated in suspension with 100 ng/mL LPS for 5–10 min. The cells were then transferred at 100,000 cells per well into 12-well plates containing a 1:200 diluted conditioned medium with *n* = 4 for each condition. After 16 h, the medium was collected from the macrophage cultures and centrifuged at 500× *g* for 5 min. The processed medium was used to measure macrophage production of the pro-inflammatory cytokine TNFα and anti-inflammatory factor IL-10. Cytokine levels were assessed using ELISA kits (R&D Systems) according to the manufacturers suggestions, as described previously [26,28,30,41].

### 2.8. Statistical Methods

Data are expressed as mean ± SD and were analyzed using GraphPad Prism (8.2.0., GraphPad Software, San Diego, CA, USA)). Data from three or more groups were analyzed using a one-way ANOVA. The Tukey’s post hoc test was used to assess statistical significance between groups. Statistical significance was defined as ns, *p* ≥ 0.05; *, *p* < 0.05; **, *p* < 0.01; and ***, *p* < 0.001, unless otherwise indicated.

## 3. Results

### 3.1. MSC Proliferation Rate Declines When Cultured Extensively

**Figure 1:**
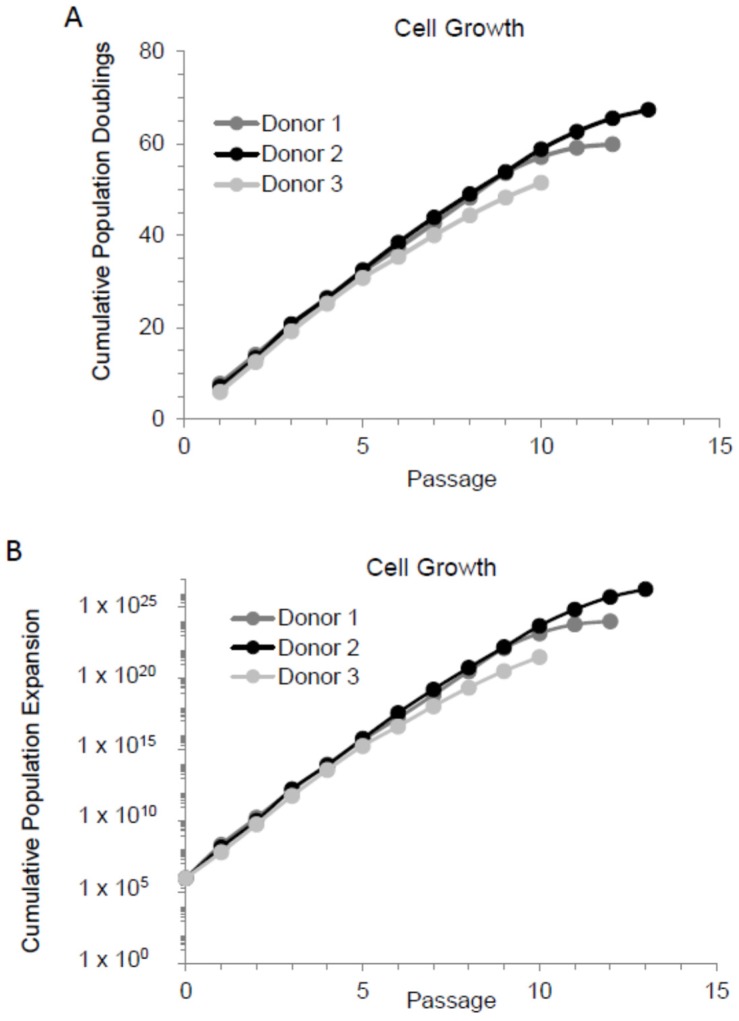
Mesenchymal stem cells (MSCs) produce up to 10^25^ cells from a small bone marrow aspirate. MSCs from three different donors were cultured in low density and passaged every seven days. Cells were counted and seeded in a low density for the next passage. This was repeated until the cell number did not double during the seven days growth. (**A**) Cell growth as cumulative population doublings. (**B**) Cell growth as cumulative population expansion.

Previous studies have demonstrated that when MSCs were extensively cultured (i.e., passaged continuously), they entered senescence [20,22,42]. In here, we cultured MSCs in 2D as adherent monolayer cultures starting from a low density (100 cells/cm^2^) and continuously passaged them every seven days. All three MSC preparations (MSC donors) used showed a rapid decline in proliferation rate after passage 7, 8, or 9 and less than one population doubling per seven days of culture at passage 10, 12, or 13, as demonstrated by the growth curves (Figure 1A). To better appreciate how the culture expansion of a small (1 mL) bone marrow aspirate affects cell yields for clinical use, we generated a cumulative population expansion graph. A conservative estimation was made that at the end of passage 0—when the initial bone marrow culture reached approximately 70% confluency, 10^6^ MSCs existed. Using this estimation, the minimally expanded MSCs in passage 2 or 3 provided approximately 10^10^ cells, while MSCs in passages 7 and 8 provided nearly 10^20^ cells. The final achievable cell yield could be up to 10^25^ for donor 2, as shown in Figure 1B. Importantly, since the proliferation rate of MSCs appeared to decline rapidly after passages 7–9, for the purposes of this study, cells expanded to passage 7–9 are considered as extensively expanded.

### 3.2. Spheroids Generated from Early Passage and Extensively Expanded MSCs Demonstrate Similar Gene Expression Patterns

**Figure 2:**
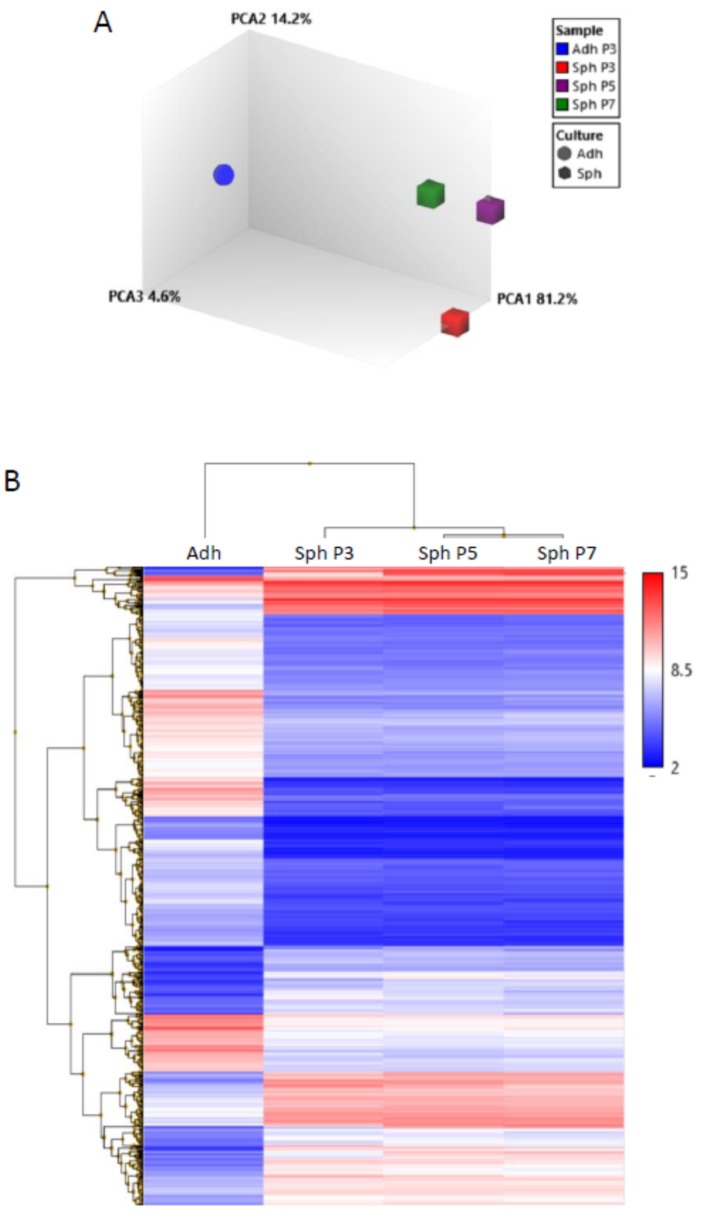
Gene expression is similar in spheroids generated from both extensively expanded and early passage MSCs. Spheroids were generated from passage 3, 5, and 7 MSCs and employed for gene expression microarrays. (**A**) Principal component analysis of the microarray data. Cube characters are spheroid MSCs from different passages and ball character is adherent monolayer MSCs. (**B**) Hierarchical clustering of the differentially expressed genes between adherent monolayer MSCs and spheroid MSCs from different passages. Red color indicates a high gene expression, and blue color indicates a low gene expression. Scale demonstrates log_2_ gene expression value. Abbreviations: Adh, adherent monolayer MSCs; PCA1, principal component 1; P3, passage 3; Sph, spheroid MSCs.

**Figure 3:**
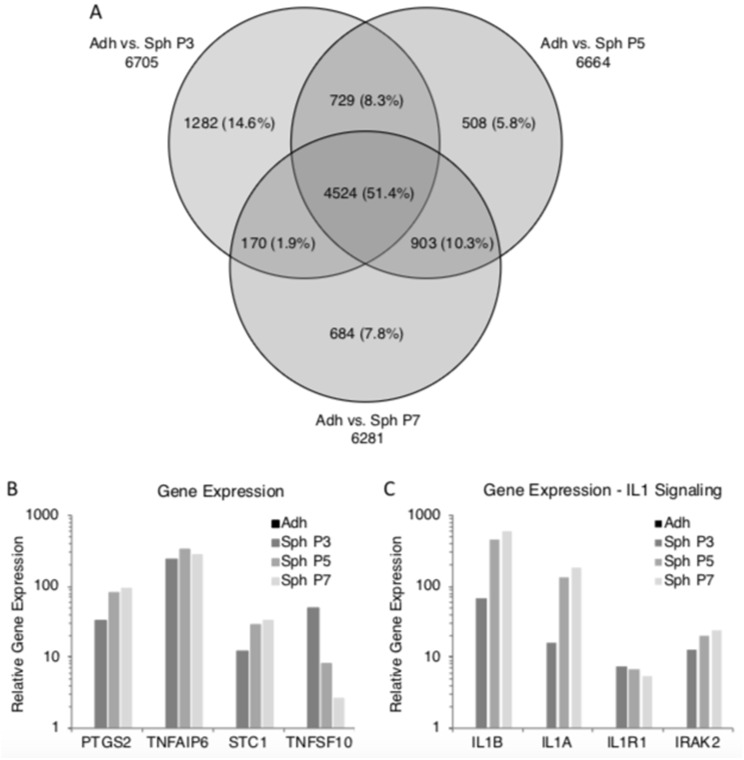
Spheroids generated from extensively expanded MSCs maintain high expression of potentially therapeutic molecules. Gene expression data were searched for differentially expressed genes between adherent monolayer MSCs and spheroids from various passages. Furthermore, the expression of potentially therapeutic genes and IL-1 signaling molecules were queried from the microarray data. (**A**) Venn diagram of differentially expressed genes. (**B**) Expression of potentially therapeutic genes in spheroid MSCs. (**C**) Expression of IL-1 signaling related genes in spheroid MSCs. Abbreviations: Adh, adherent monolayer MSCs; P3, passage 3; Sph, spheroid MSCs.

In our previous studies, we demonstrated that MSCs grown in hanging drops aggregated into spheroids [25,26,27,28]. However, previous studies of 3D cultured MSCs had focused on minimally expanded, or early passage cells, whereas in some therapeutic instances, the use of later passage cultures might be necessary. In here, we generated MSC spheroids in hanging drops from adherent monolayer MSCs at passages 3, 5, and 7, and we studied their gene expression profiles with microarrays. Principal component analysis demonstrated that gene expression levels of MSC spheroids generated from all three passages were very similar to each other (Figure 2A). This was further supported by hierarchical clustering of the differentially expressed genes between the spheroids and adherent monolayer MSCs (Figure 2B). A Venn diagram of differentially expressed genes demonstrated that over 50% of these genes were shared by spheres from all three passages (Figure 3A). Spheroids from each passage showed some genes that were differentially expressed in only that passage; however, the number of genes was only 6–15% (Figure 3A). The microarray data suggested that passage 5 and 7 MSC spheroids were slightly more similar to each other than to P3 spheroids, but they still shared many gene expression patterns with the P3 spheroids (Figure 2 and Figure 3A).

A further analysis of the genes differentially expressed between the adherent monolayer MSCs and spheroid MSCs was conducted using WikiPathways. Many of the differentially expressed genes were in pathways relating to metabolism of various biomolecules including glycosaminoglycans, pyrimidines, sphingolipids, glycerophospholipids, amino acids, and arachidonic acid (Supplemental Table S2). These results suggested that the MSCs were changing their metabolism preferences as they aggregated into spheroids. Furthermore, pathways relating to cell cycle, adhesion, and extracellular matrix were significantly represented in the differentially expressed genes, suggesting modifications in the cell-to-cell and cell-to-matrix interactions as the MSCs assembled into spheroids (Supplemental Table S2). In addition, various cell signaling pathways, such as IL-1 and senescence in cancer and the senescence-associated secretory phenotype (SASP), were significantly presented in the differentially expressed genes, thus suggesting major changes in cell communication (Supplemental Table S2). Many of the genes up-regulated or down-regulated in the spheroids had very similar fold changes in all passages when compared to the adherent monolayer MSCs. (Supplemental Table S3).

### 3.3. Spheroids Generated from Extensively Expanded MSCs Maintain the High Expression of Potentially Therapeutic Genes

To study if the spheroids generated from extensively expanded MSCs still expressed high levels of potentially therapeutic genes that we identified previously [26,28], we searched the microarray data for their gene expressions. Microarrays suggested that PTGS2 (COX2), TNFAIP6 (TSG6), STC1, and TNSF10 (TRAIL) were all still highly expressed in spheroids generated from P5 and P7 MSCs (Figure 3B) when compared to the adherent monolayer MSCs. Microarray data suggested that PTGS2, TNFAIP6, and STC1 were even more up-regulated in spheroids generated from late passage cells than spheroids from early passage when compared to the adherent monolayer MSCs (Figure 3B). However, as suggested by the microarray data, the high TNSF10 expression got smaller as the MSCs were passaged but was still up-regulated in P7 spheroids when compared to the adherent monolayer MSCs (Figure 3B).

We previously demonstrated the importance of IL-1 signaling in the up-regulation of the potentially therapeutic genes in MSC spheroids [27]. Therefore, the microarray data were queried for the key IL-1 signaling related genes. The microarray data suggested that the up-regulation of IL-1B, IL-1A, IL-1R1, and IRAK2 were maintained in spheroids generated from all passages (Figure 3C).

### 3.4. MSC Spheroids Generated from Extensively Expanded Cells Secrete High Amounts of Anti-Inflammatory/Immunomodulatory Factors

**Figure 4:**
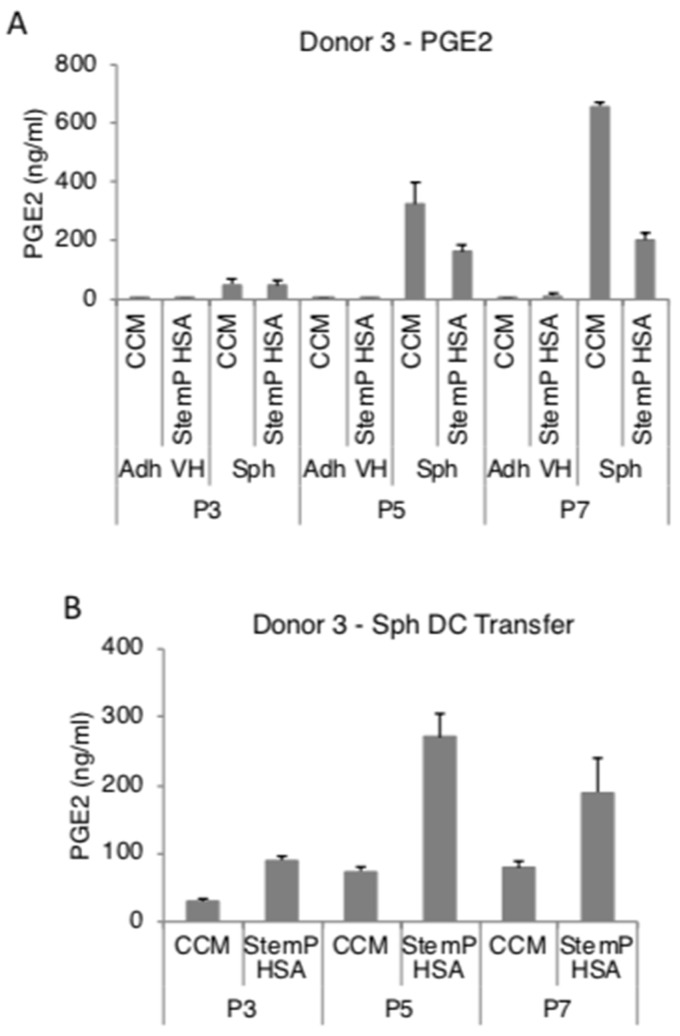
Spheroids generated from extensively expanded MSCs secrete high amounts of prostaglandin E2 (PGE2). MSCs from various passages (3, 5, and 7) were cultured as spheroids and as very high density monolayers in fetal bovine serum (FBS)-containing and xeno-free media. The conditioned medium was harvested for PGE2 ELISA. Spheroids were also dissociated and the resulting cells were tested for their ability to maintain PGE2 secretion. (**A**) PGE2 secretion by spheroids and very high density monolayer MSCs in FBS-containing media and xeno-free media at different passages. (**B**) PGE2 secretion by spheroid-derived cells from FBS-containing media and xeno-free media at different passages. Abbreviations: Adh VH, adherent very high density monolayer MSCs; CCM, complete culture medium; P3, passage 3; Sph, spheroid MSCs; Sph DC, spheroid-derived MSCs; StemP HSA, StemPro xeno-free media with human serum albumin.

**Figure 5:**
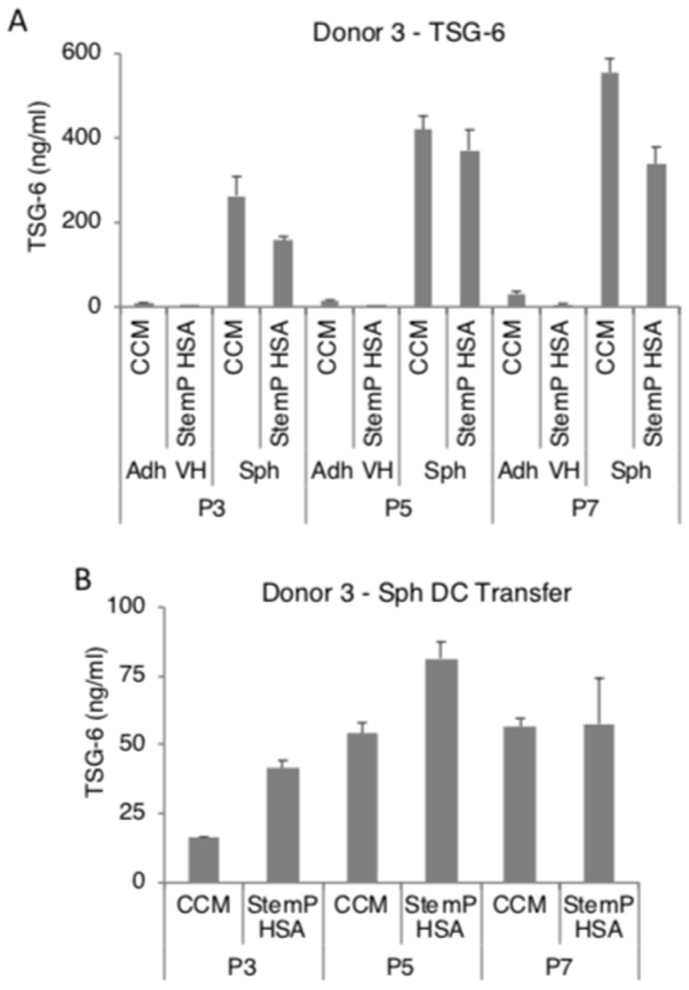
Spheroids generated from extensively expanded MSCs secrete high amounts of tumor necrosis factor-stimulated gene 6 (TSG-6). MSCs from various passages (3, 5, and 7) were cultured as spheroids and as very high density monolayers in FBS-containing and xeno-free media. The conditioned medium was harvested for TSG-6 ELISA. Spheroids were also dissociated and the resulting cells were tested for their ability to maintain TSG-6 secretion. (**A**) TSG-6 secretion by spheroids and very high density monolayer MSCs in FBS-containing media and xeno-free media at different passages. (**B**) TSG-6 secretion by spheroid-derived cells from FBS-containing media and xeno-free media at different passages. Abbreviations: Adh VH, adherent very high density monolayer MSCs; CCM, complete culture medium; P3, passage 3; Sph, spheroid MSCs; Sph DC, spheroid-derived MSCs; StemP HSA, StemPro xeno-free media with human serum albumin.

**Figure 6:**
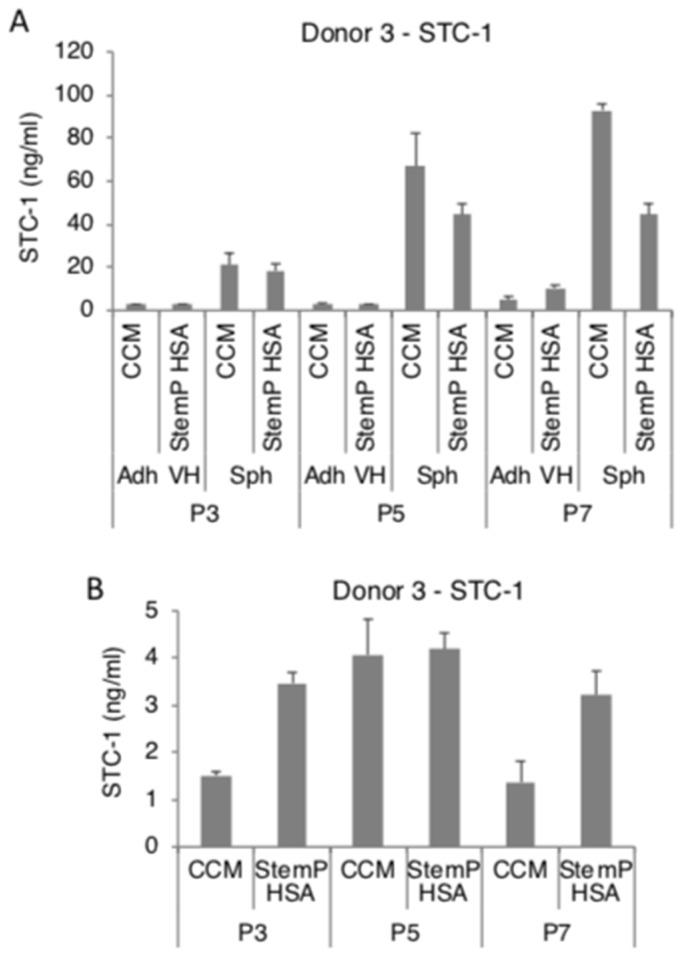
Spheroids generated from extensively expanded MSCs secrete high amounts of stanniocalcin 1 (STC-1). MSCs from various passages (3, 5, and 7) were cultured as spheroids and as very high density monolayers in FBS-containing and xeno-free media. The conditioned medium was harvested for STC-1 ELISA. Spheroids were also dissociated and the resulting cells were tested for their ability to maintain STC-1 secretion. (**A**) STC-1 secretion by spheroids and very high density monolayer MSCs in FBS-containing media and xeno-free media at different passages. (**B**) STC-1 secretion by spheroid-derived cells from FBS-containing media and xeno-free media at different passages. Abbreviations: Adh VH, adherent very high density monolayer MSCs; CCM, complete culture medium; P3, passage 3; Sph, spheroid MSCs; Sph DC, spheroid-derived MSCs; StemP HSA, StemPro xeno-free media with human serum albumin.

We had previously reported that MSC spheroids secreted high amounts of PGE2, TSG-6, and STC-1 in FBS-containing and in a specific xeno-free media [25,26,27,28]. In here, MSC spheroids generated from extensively expanded cells from three donors secreted high levels of PGE2 in both FBS-containing media and xeno-free media (Figure 4A, Supplemental Figures S1A and S2A). To study if the high PGE2 production was maintained at least for short term in 2D after the 3D pre-activation, the obtained MSC spheroids were enzymatically and mechanically dissociated. The resulting cells maintained a high level of secretion of PGE2 even after transfer into 2D cultures (Figure 4B, Supplemental Figures S1B and S2B). MSC spheroids also secreted high amounts of TSG-6 (Figure 5A, Supplemental Figures S3A and S4A) and STC-1 (Figure 6A, Supplemental Figures S5A and S6A) in media supplemented with FBS or in the xeno-free medium. The secretion of TSG-6 (Figure 5B, Supplemental Figures S3B and S4B) and STC-1 (Figure 6B, Supplemental Figures S5B and S6B) was also maintained in 2D in cells dissociated from spheroids generated in a serum containing media and xeno-free conditions.

### 3.5. MSC Spheroids Generated from Extensively Expanded Cells Maintain Their Ability to Suppress Stimulated Macrophages

**Figure 7:**
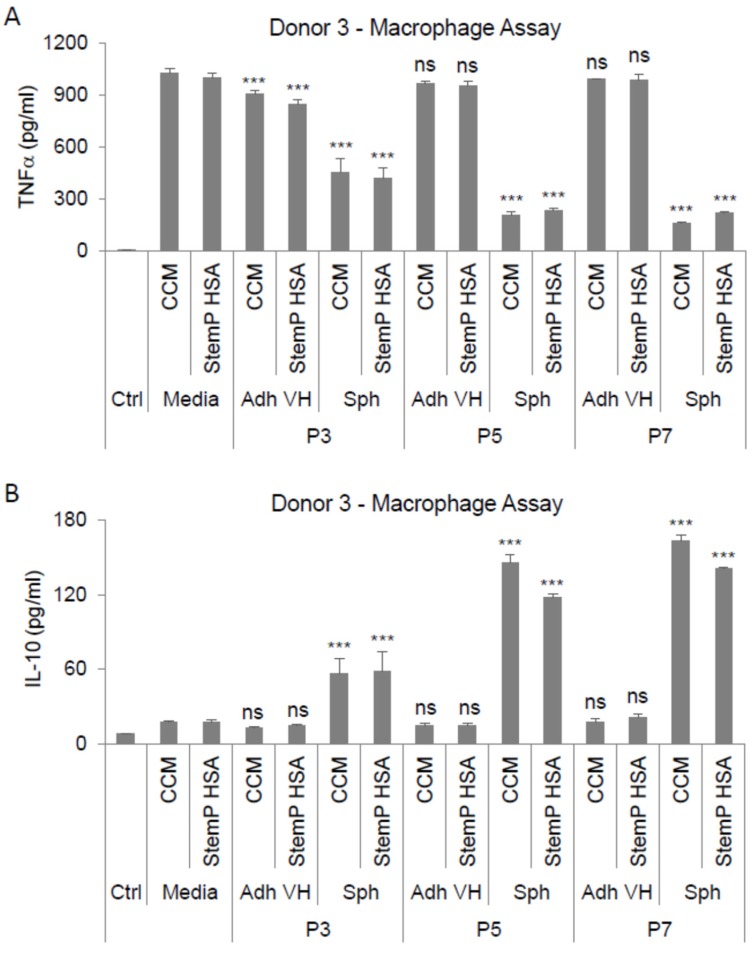
Spheroids generated from extensively expanded MSCs exhibit anti-inflammatory properties. MSCs from various passages (3, 5, and 7) were cultured as spheroids and as very high density monolayers in FBS-containing and xeno-free media. Conditioned media from these cultures were applied on lipopolysaccharide (LPS)-stimulated macrophages and anti-inflammatory effects were studied. (**A**) Conditioned media effects on TNFα secretion by LPS-stimulated macrophages. (**B**) Conditioned media effects on IL-10 secretion by LPS-stimulated macrophages. Compared to the appropriate media control (i.e., CCM or StemP HSA). Statistical significance was defined as ns, *p* ≥ 0.05; and ***, *p* < 0.001, Abbreviations: Adh VH, adherent very high density monolayer MSCs; CCM, complete culture medium; Ctrl, control unstimulated macrophages; P3, passage 3; Sph, spheroid MSCs; StemP HSA, StemPro xeno-free media with human serum albumin.

In our previous studies, we demonstrated that 3D pre-activated MSCs showed anti-inflammatory effects both in vitro and in vivo [25,26,27,28]. More specifically, we showed that MSC spheroids could suppress LPS-stimulated macrophages primarily through the secretion of PGE2 [28]. Since the spheroids from extensively expanded MSCs still secreted high amounts of PGE2, their ability to suppress LPS-activate macrophages was studied. Spheroids from both the FBS-containing cultures and the xeno-free cultures secreted factors that suppressed the secretion of the pro-inflammatory cytokine TNF-α (Figure 7A) and increased the secretion of the anti-inflammatory cytokine IL-10 (Figure 7B) by the stimulated macrophages. Higher macrophage suppression was achieved by the conditioned medium from the later passage cultures (Figure 7)

## 4. Discussion

MSCs are the progenitor cells of most tissues that have been studied in detail both in pre-clinical and clinical settings [1,4,7,13,43,44]. Common to many MSC studies is the culture of MSCs in 2D under xenogeneic conditions (i.e., conditions that involve tissues/cells belonging to a different species) and the extensive expansion of the cells. These conditions can change the MSC characteristics and cause problems when transferring basic research into the clinical setting [16,45,46]. Here, we demonstrated culture conditions that promoted the activation of extensively expanded MSCs in FBS-containing and xeno-free conditions. Furthermore, we showed that the potential anti-inflammatory effects of the MSC spheroids and spheroid-derived cells are maintained in MSCs even after extensive culture expansion.

The culture of cells in 2D on tissue culture plastic is the most common and convenient method to expand cells such as MSCs [4]. MSCs are easily expandable under these conditions, especially when the culture media contains FBS. However, 2D cultures do not mimic the in vivo niches of MSCs well and may therefore change the cells and affect their usability [19,47]. Many of the changes have been attributed to the lack of cell-to-cell contacts and the requirement of the use of enzymes and other chemicals to break the tight binding between the cells and the plastic, which can lead into further change and even damage of the cells. In 3D, cells are able to communicate with neighboring cells better, and cell-to-cell and cell-to-matrix connections are formed easily [19,47]. MSCs and many other cells spontaneously aggregate into 3D spheroids when culture under conditions in which plastic adherence is not permitted [19,48]. The aggregation can be achieved through culture in hanging drops, as demonstrated here, but also through other means such as non-adherent culture dishes, rotating wall vessels, and precise printing techniques [19,49]. Hanging drop cultures are somewhat labor intensive, but they are inexpensive and allow for the formation of uniform sized spheroids through easy manipulation of the drop volume and cell concentration. We previously demonstrated that MSCs aggregate into spheroids in hanging drops even under xeno-free conditions and activate anti-inflammatory and immunomodulatory factors similarly to the FBS-containing hanging drop cultures [25,26,27,28].

FBS is a common, easy to obtain, and relatively inexpensive media additive that provides ample nutrients to growing cells. However, manufactured lots of FBS do not always work the same and add xenogeneic molecules that can be internalized by the cells [50]. Xenogeneic molecules can act as antigens when the cells are delivered into the patients and can therefore generate undesired immune reactions, thus hampering the therapeutic use of the cells that have been expanded with FBS-containing media [51]. In here, we continued our previous research regarding xeno-free 3D cultures and expanded the studies using extensively cultured MSCs [25].

To obtain the large numbers of MSCs for therapeutic use, the cells must be extensively expanded in culture [16,17]. As many research experiments typically use low-passage MSCs, many unknowns persist regarding the characteristics and usability of late passage MSCs. However, some studies have shown that MSCs enter replicative senescence after extensive culture and change their transcriptome, leading into morphological and functional changes of the cells [16,21,42,52]. In here, we showed that the MSC proliferation rate steadily declined following extensive expansion, suggesting the cells entered senescence at various times, as supported by previous studies [42,52]. Throughout the extensive expansion of the MSCs in the current study, cells were pre-activated in 3D for the study of the transcriptomes and functional characteristics of the cells. Our results showed that the spheroids generated from the MSCs at different passages were transcriptionally very similar and much closer to each other than they were to the 2D cultured MSCs. This was somewhat surprising, as our study employed MSCs from various donors and different passages, including late passage cells showing a decline in proliferation rate. The results suggested that even a short 3D culture of 72 h was able to change the characteristics of extensively expanded MSCs comparable to the early passage 3D cultures.

Our previous research had demonstrated the importance of up-regulated IL-1 signaling in 3D MSC spheroids to the potential anti-inflammatory effects of the cells [27]. Here, spheroids generated from different passage MSCs exhibited an increased expression of the IL-1 signaling molecules important in activating the expression of the observed anti-inflammatory and immunomodulatory related factors PTGS2 (COX-2), TNFAIP6 (TSG-6), and STC1. These results suggested that the short 3D culture was able to activate the IL-1 signaling that controlled the expression of the aforementioned factors. Furthermore, our results showed that the ability of 3D-culture-activated MSCs to secrete high levels of PGE2, TSG-6, and STC-1 was maintained regardless of the passage of the 2D expanded cells used to generate the spheroids. These findings were also demonstrated under xeno-free conditions using the optimal FBS-free media formulation identified in our previous studies [25]. In addition to IL-1, several other inflammatory cytokines/pathways were upregulated by spheroid MSCs, corroborating our data in prior reports [25,26,27,28]. Some of these inflammatory factors are components of the senescence-associate secretory phenotype (SASP), a secretory program rich in cytokines and proteases that allow senescent cells to contribute to a variety of physiological and pathological processes [53]. The critical role for the SASP cytokine IL-1, and perhaps other SASP factors, in regards to MSC spheroid functionality is in agreement with the concept that pro-inflammatory cytokines and other tissue-derived injury signals provide a critical stimulus for the MSC production of immune-modulating agents [6,9,10,11,12,13]. MSC-derived cytokines, such as IL-6 and IL-8, have been shown to help perpetuate the PGE2-modulating signal [54]. In addition, specific cytokines/chemokines produced by MSCs have been suggested to attract monocytes and other inflammatory/immune cells, thus allowing MSCs to target their immune-modulating activities [55]. We have also recently shown that the SASP program could be important for spheroid MSCs to promote tumor dormancy in breast cancer [56]. Taken together, the assortment of SASP factors produced by activated MSCs could provide the cells with the tools needed to exert diverse functionality across of spectrum of physiological and pathological processes, including the concept that spheroid MSCs have both immune-modulating and anti-cancer effects.

To compare the various MSC preparations further, we conducted in vitro functional studies. In these studies, the 3D activated MSCs showed significant anti-inflammatory effects in an in vitro stimulated macrophage system. The conditioned media from the 3D MSC cultures suppressed the secretion of the pro-inflammatory cytokine TNF-α while increasing the secretion of the anti-inflammatory cytokine IL-10 by the stimulated macrophages. These results suggested that the extensively expanded MSCs can be pre-activated in 3D cultures to secrete functional anti-inflammatory molecules. Further research needs to be completed to test if the anti-cancer effects of the MSC spheroids are maintained throughout the passaging process. Additionally, further studies are required for the testing of the in vivo efficacy of the 3D spheroids generated from extensively expanded MSCs.

## 5. Conclusions

In this study, we showed a process to pre-activate extensively expanded MSCs through 3D culturing to express and secrete important anti-inflammatory and immunomodulatory factors. Furthermore, we demonstrated that the spheroids derived from various stages of the culture expansion were very similar and were able to suppress stimulated macrophages in vitro. This study highlights the possibility of activating otherwise less useable MSC preparations generating large numbers of potentially therapeutic MSCs.

## Supplementary Materials

The following are available online at https://www.mdpi.com/2073-4409/8/9/1031/s1, Figure S1: Cell surface marker expression for a representative MSC donor. Table S1: Expression of the common MSC markers for the donors in this study. Table S2: Selected WikiPathways enriched in differentially expressed genes between adherent monolayer and spheroid cultures of MSCs. Table S3: The top 20 up-regulated and down-regulated genes in MSC spheroids from different passages compared to adherent monolayer MSCs (fold change). Figure S2: Spheroids generated from extensively expanded MSCs from donor 1 secrete high amounts of PGE2. Spheroids and very high density monolayer MSCs were cultured in FBS-containing media and in xeno-free media from MSCs after passages 3, 5, 7, and 9. The conditioned medium was harvested after three days for PGE2 ELISA. Spheroids were also dissociated, and the resulting cells were tested for their ability to maintain PGE2 secretion. (A) PGE2 secretion by spheroids and very high density monolayer MSCs in FBS-containing media and xeno-free media at different passages. (B) PGE2 secretion by spheroid-derived cells from FBS-containing media and xeno-free media at different passages. Figure S3: Spheroids generated from extensively expanded MSCs from donor 2 secrete high amounts of PGE2. Spheroids and very high density monolayer MSCs were cultured in FBS-containing media and in xeno-free media from MSCs after passages 3, 5, and 7. The conditioned medium was harvested after three days for PGE2 ELISA. Spheroids were also dissociated, and the resulting cells were tested for their ability to maintain PGE2 secretion. (A) PGE2 secretion by spheroids and very high density monolayer MSCs in FBS-containing media and xeno-free media at different passages. (B) PGE2 secretion by spheroid-derived cells from FBS-containing media and xeno-free media at different passages. Figure S4: Spheroids generated from extensively expanded MSCs from donor 1 secrete high amounts of TSG-6. Spheroids and very high density monolayer MSCs were cultured in FBS-containing media and in xeno-free media from MSCs after passages 3, 5, 7, and 9. The conditioned medium was harvested after three days for TSG-6 ELISA. Spheroids were also dissociated, and the resulting cells were tested for their ability to maintain TSG-6 secretion. (A) TSG-6 secretion by spheroids and very high density monolayer MSCs in FBS-containing media and xeno-free media at different passages. (B) TSG-6 secretion by spheroid-derived cells from FBS-containing media and xeno-free media at different passages. Figure S5: Spheroids generated from extensively expanded MSCs from donor 2 secrete high amounts of TSG-6. Spheroids and very high density monolayer MSCs were cultured in FBS-containing media and in xeno-free media from MSCs after passages 3, 5, and 7. The conditioned medium was harvested after three days for TSG-6 ELISA. Spheroids were also dissociated, and the resulting cells were tested for their ability to maintain TSG-6 secretion. (A) TSG-6 secretion by spheroids and very high density monolayer MSCs in FBS-containing media and xeno-free media at different passages. (B) TSG-6 secretion by spheroid-derived cells from FBS-containing media and xeno-free media at different passages. Figure S6: Spheroids generated from extensively expanded MSCs from donor 1 secrete high amounts of STC-1. Spheroids and very high density monolayer MSCs were cultured in FBS-containing media and in xeno-free media from MSCs after passages 3, 5, 7, and 9. The conditioned medium was harvested after three days for STC-1 ELISA. Spheroids were also dissociated, and the resulting cells were tested for their ability to maintain STC-1 secretion. (A) STC-1 secretion by spheroids and very high density monolayer MSCs in FBS-containing media and xeno-free media at different passages. (B) STC-1 secretion by spheroid-derived cells from FBS-containing media and xeno-free media at different passages. Figure S7: Spheroids generated from extensively expanded MSCs from donor 2 secrete high amounts of STC-1. Spheroids and very high density monolayer MSCs were cultured in FBS-containing media and in xeno-free media from MSCs after passages 3, 5, and 7. The conditioned medium was harvested after three days for STC-1 ELISA. Spheroids were also dissociated, and the resulting cells were tested for their ability to maintain STC-1 secretion. (A) STC-1 secretion by spheroids and very high density monolayer MSCs in FBS-containing media and xeno-free media at different passages. (B) STC-1 secretion by spheroid-derived cells from FBS-containing media and xeno-free media at different passages.

## References

[1] Spees J.L., Lee R.H., Gregory C.A. (2016). Mechanisms of mesenchymal stem/stromal cell function. Stem Cell Res. Ther..

[2] Caplan A.I. (1991). Mesenchymal stem cells. J. Orthop. Res..

[3] Pittenger M.F., Mackay A.M., Beck S.C., Jaiswal R.K., Douglas R., Mosca J.D., Moorman M.A., Simonetti D.W., Craig S., Marshak D.R. (1999). Multilineage potential of adult human mesenchymal stem cells. Science.

[4] Dominici M., Le Blanc K., Mueller I., Slaper-Cortenbach I., Marini F., Krause D., Deans R., Keating A., Prockop D., Horwitz E. (2006). Minimal criteria for defining multipotent mesenchymal stromal cells. The International Society for Cellular Therapy position statement. Cytotherapy.

[5] Prockop D.J. (1997). Marrow stromal cells as stem cells for nonhematopoietic tissues. Science.

[6] Ankrum J., Karp J.M. (2010). Mesenchymal stem cell therapy: Two steps forward, one step back. Trends Mol. Med..

[7] da Silva Meirelles L., Chagastelles P.C., Nardi N.B. (2006). Mesenchymal stem cells reside in virtually all post-natal organs and tissues. J. Cell Sci..

[8] Crisan M., Yap S., Casteilla L., Chen C.W., Corselli M., Park T.S., Andriolo G., Sun B., Zheng B., Zhang L. (2008). A perivascular origin for mesenchymal stem cells in multiple human organs. Cell Stem Cell.

[9] Ma S., Xie N., Li W., Yuan B., Shi Y., Wang Y. (2014). Immunobiology of mesenchymal stem cells. Cell Death Differ..

[10] Uccelli A., de Rosbo N.K. (2015). The immunomodulatory function of mesenchymal stem cells: Mode of action and pathways. Ann. N. Y. Acad. Sci..

[11] Ghannam S., Bouffi C., Djouad F., Jorgensen C., Noël D. (2010). Immunosuppression by mesenchymal stem cells: Mechanisms and clinical applications. Stem Cell Res. Ther..

[12] Gebler A., Zabel O., Seliger B. (2012). The immunomodulatory capacity of mesenchymal stem cells. Trends Mol. Med..

[13] Keating A. (2012). Mesenchymal stromal cells: New directions. Cell Stem Cell.

[14] Squillaro T., Peluso G., Galderisi U. (2016). Clinical Trials With Mesenchymal Stem Cells: An Update. Cell Transplant.

[15] Samsonraj R.M., Raghunath M., Nurcombe V., Hui J.H., van Wijnen A.J., Cool S.M. (2017). Concise Review: Multifaceted Characterization of Human Mesenchymal Stem Cells for Use in Regenerative Medicine. Stem Cells Transl. Med..

[16] Hoch A.I., Leach J.K. (2015). Concise review: Optimizing expansion of bone marrow mesenchymal stem/stromal cells for clinical applications. Stem Cells Transl. Med..

[17] Stolzing A., Jones E., McGonagle D., Scutt A. (2008). Age-related changes in human bone marrow-derived mesenchymal stem cells: Consequences for cell therapies. Mech. Ageing Dev..

[18] Friedenstein A.J., Gorskaja J.F., Kulagina N.N. (1976). Fibroblast precursors in normal and irradiated mouse hematopoietic organs. Exp. Hematol..

[19] Cesarz Z., Tamama K. (2016). Spheroid Culture of Mesenchymal Stem Cells. Stem Cells Int..

[20] Banfi A., Muraglia A., Dozin B., Mastrogiacomo M., Cancedda R., Quarto R. (2000). Proliferation kinetics and differentiation potential of ex vivo expanded human bone marrow stromal cells: Implications for their use in cell therapy. Exp. Hematol..

[21] Kretlow J.D., Jin Y.Q., Liu W., Zhang W.J., Hong T.H., Zhou G., Baggett L.S., Mikos A.G., Cao Y. (2008). Donor age and cell passage affects differentiation potential of murine bone marrow-derived stem cells. BMC Cell Biol..

[22] Sepúlveda J.C., Tomé M., Fernández M.E., Delgado M., Campisi J., Bernad A., González M.A. (2014). Cell senescence abrogates the therapeutic potential of human mesenchymal stem cells in the lethal endotoxemia model. Stem Cells.

[23] Galipeau J. (2013). The mesenchymal stromal cells dilemma--does a negative phase III trial of random donor mesenchymal stromal cells in steroid-resistant graft-versus-host disease represent a death knell or a bump in the road?. Cytotherapy.

[24] Moll G., Rasmusson-Duprez I., von Bahr L., Connolly-Andersen A.M., Elgue G., Funke L., Hamad O.A., Lönnies H., Magnusson P.U., Sanchez J. (2012). Are therapeutic human mesenchymal stromal cells compatible with human blood?. Stem Cells.

[25] Ylostalo J.H., Bartosh T.J., Tiblow A., Prockop D.J. (2014). Unique characteristics of human mesenchymal stromal/progenitor cells pre-activated in 3-dimensional cultures under different conditions. Cytotherapy.

[26] Bartosh T.J., Ylöstalo J.H., Mohammadipoor A., Bazhanov N., Coble K., Claypool K., Lee R.H., Choi H., Prockop D.J. (2010). Aggregation of human mesenchymal stromal cells (MSCs) into 3D spheroids enhances their antiinflammatory properties. Proc. Natl. Acad. Sci. USA.

[27] Bartosh T.J., Ylöstalo J.H., Bazhanov N., Kuhlman J., Prockop D.J. (2013). Dynamic compaction of human mesenchymal stem/precursor cells into spheres self-activates caspase-dependent IL1 signaling to enhance secretion of modulators of inflammation and immunity (PGE2, TSG6, and STC1). Stem Cells.

[28] Ylöstalo J.H., Bartosh T.J., Coble K., Prockop D.J. (2012). Human mesenchymal stem/stromal cells cultured as spheroids are self-activated to produce prostaglandin E2 that directs stimulated macrophages into an anti-inflammatory phenotype. Stem Cells.

[29] Bazhanov N., Ylostalo J.H., Bartosh T.J., Tiblow A., Mohammadipoor A., Foskett A., Prockop D.J. (2016). Intraperitoneally infused human mesenchymal stem cells form aggregates with mouse immune cells and attach to peritoneal organs. Stem Cell Res. Ther..

[30] Ylostalo J.H., Bazhanov N., Mohammadipoor A., Bartosh T.J. (2017). Production and Administration of Therapeutic Mesenchymal Stem/Stromal Cell (MSC) Spheroids Primed in 3-D Cultures Under Xeno-free Conditions. J. Vis. Exp..

[31] Lee R.H., Pulin A.A., Seo M.J., Kota D.J., Ylostalo J., Larson B.L., Semprun-Prieto L., Delafontaine P., Prockop D.J. (2009). Intravenous hMSCs improve myocardial infarction in mice because cells embolized in lung are activated to secrete the anti-inflammatory protein TSG-6. Cell Stem Cell.

[32] Choi H., Lee R.H., Bazhanov N., Oh J.Y., Prockop D.J. (2011). Anti-inflammatory protein TSG-6 secreted by activated MSCs attenuates zymosan-induced mouse peritonitis by decreasing TLR2/NF-κB signaling in resident macrophages. Blood.

[33] Danchuk S., Ylostalo J.H., Hossain F., Sorge R., Ramsey A., Bonvillain R.W., Lasky J.A., Bunnell B.A., Welsh D.A., Prockop D.J. (2011). Human multipotent stromal cells attenuate lipopolysaccharide-induced acute lung injury in mice via secretion of tumor necrosis factor-α-induced protein 6. Stem Cell Res. Ther..

[34] Foskett A.M., Bazhanov N., Ti X., Tiblow A., Bartosh T.J., Prockop D.J. (2014). Phase-directed therapy: TSG-6 targeted to early inflammation improves bleomycin-injured lungs. Am. J. Physiol. Lung Cell Mol. Physiol..

[35] Mohammadipoor A., Lee R.H., Prockop D.J., Bartosh T.J. (2016). Stanniocalcin-1 attenuates ischemic cardiac injury and response of differentiating monocytes/macrophages to inflammatory stimuli. Transl. Res..

[36] Oh J.Y., Roddy G.W., Choi H., Lee R.H., Ylöstalo J.H., Rosa R.H., Prockop D.J. (2010). Anti-inflammatory protein TSG-6 reduces inflammatory damage to the cornea following chemical and mechanical injury. Proc. Natl. Acad. Sci. USA.

[37] Roddy G.W., Oh J.Y., Lee R.H., Bartosh T.J., Ylostalo J., Coble K., Rosa R.H., Prockop D.J. (2011). Action at a distance: Systemically administered adult stem/progenitor cells (MSCs) reduce inflammatory damage to the cornea without engraftment and primarily by secretion of TNF-α stimulated gene/protein 6. Stem Cells.

[38] Roddy G.W., Rosa R.H., Oh J.Y., Ylostalo J.H., Bartosh T.J., Choi H., Lee R.H., Yasumura D., Ahern K., Nielsen G. (2012). Stanniocalcin-1 rescued photoreceptor degeneration in two rat models of inherited retinal degeneration. Mol. Ther..

[39] Song H.B., Park S.Y., Ko J.H., Park J.W., Yoon C.H., Kim D.H., Kim J.H., Kim M.K., Lee R.H., Prockop D.J. (2018). Mesenchymal Stromal Cells Inhibit Inflammatory Lymphangiogenesis in the Cornea by Suppressing Macrophage in a TSG-6-Dependent Manner. Mol. Ther..

[40] Xie J., Broxmeyer H.E., Feng D., Schweitzer K.S., Yi R., Cook T.G., Chitteti B.R., Barwinska D., Traktuev D.O., Van Demark M.J. (2015). Human adipose-derived stem cells ameliorate cigarette smoke-induced murine myelosuppression via secretion of TSG-6. Stem Cells.

[41] Bartosh T.J., Ylostalo J.H. (2014). Macrophage Inflammatory Assay. Bio. Protoc..

[42] Larson B.L., Ylostalo J., Lee R.H., Gregory C., Prockop D.J. (2010). Sox11 is expressed in early progenitor human multipotent stromal cells and decreases with extensive expansion of the cells. Tissue Eng. Part A.

[43] Le Blanc K., Davies L.C. (2018). MSCs-cells with many sides. Cytotherapy.

[44] Uccelli A., Moretta L., Pistoia V. (2008). Mesenchymal stem cells in health and disease. Nat. Rev. Immunol..

[45] Dimarino A.M., Caplan A.I., Bonfield T.L. (2013). Mesenchymal stem cells in tissue repair. Front. Immunol..

[46] Kouroupis D., Sanjurjo-Rodriguez C., Jones E., Correa D. (2019). Mesenchymal Stem Cell Functionalization for Enhanced Therapeutic Applications. Tissue Eng. Part B Rev..

[47] Mueller-Klieser W. (1997). Three-dimensional cell cultures: From molecular mechanisms to clinical applications. Am. J. Physiol..

[48] Sart S., Tsai A.C., Li Y., Ma T. (2014). Three-dimensional aggregates of mesenchymal stem cells: Cellular mechanisms, biological properties, and applications. Tissue Eng. Part B Rev..

[49] Achilli T.M., Meyer J., Morgan J.R. (2012). Advances in the formation, use and understanding of multi-cellular spheroids. Expert. Opin. Biol. Ther..

[50] Banfi F., Colombini A., Perucca Orfei C., Parazzi V., Ragni E. (2018). Validation of reference and identity-defining genes in human mesenchymal stem cells cultured under unrelated fetal bovine serum batches for basic science and clinical application. Stem Cell Rev..

[51] Spees J.L., Gregory C.A., Singh H., Tucker H.A., Peister A., Lynch P.J., Hsu S.C., Smith J., Prockop D.J. (2004). Internalized antigens must be removed to prepare hypoimmunogenic mesenchymal stem cells for cell and gene therapy. Mol. Ther..

[52] Drela K., Stanaszek L., Nowakowski A., Kuczynska Z., Lukomska B. (2019). Experimental Strategies of Mesenchymal Stem Cell Propagation: Adverse Events and Potential Risk of Functional Changes. Stem Cells Int..

[53] Sun Y., Coppé J.P., Lam E.W. (2018). Cellular Senescence: The Sought or the Unwanted?. Trends Mol. Med..

[54] Li H.J., Reinhardt F., Herschman H.R., Weinberg R.A. (2012). Cancer-stimulated mesenchymal stem cells create a carcinoma stem cell niche via prostaglandin E2 signaling. Cancer Discov..

[55] Kyurkchiev D., Bochev I., Ivanova-Todorova E., Mourdjeva M., Oreshkova T., Belemezova K., Kyurkchiev S. (2014). Secretion of immunoregulatory cytokines by mesenchymal stem cells. World J. Stem Cells.

[56] Bartosh T.J., Ullah M., Zeitouni S., Beaver J., Prockop D.J. (2016). Cancer cells enter dormancy after cannibalizing mesenchymal stem/stromal cells (MSCs). Proc. Natl. Acad. Sci. USA.

