# Supplementary material for: Efficacy of 3D Culture Priming is Maintained in Human Mesenchymal Stem Cells after Extensive Expansion of the Cells
Source: Cells. 2019 Sep 5;8(9):1031. doi: 10.3390/cells8091031 (PMC6770505; doi:10.3390/cells8091031)
Supplement: Supplementary file 1 [file cells-08-01031-s001.pdf]

cells

MDPI

Supplementary Figures and Tables

Supplementary Figure 1.

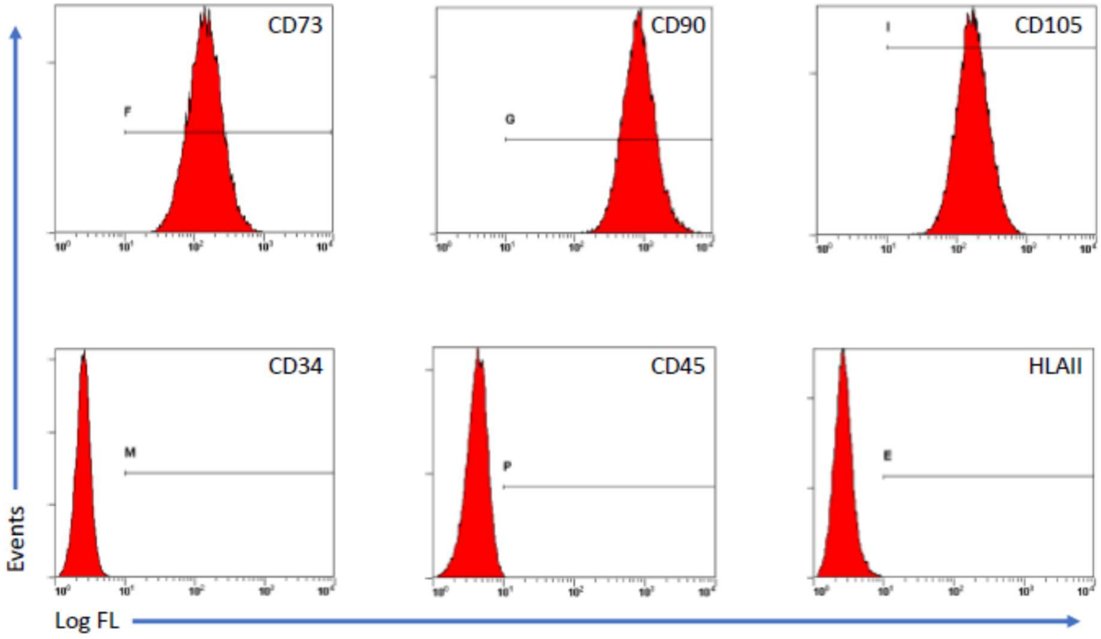

Supplementary Table 1. Expression of the common MSC markers for the donors in this study.

Markers	Donor 1	Donor 2	Donor 3
CD34/CD45/HLAII	< 1%	< 2%	< 1%
CD73	99.9%	99.7%	99.9%
CD90	99.9%	99.6%	99.9%
CD105	98.3%	99.7%	99.9%

Supplementary Table 2. Selected WikiPathways enriched in differentially expressed genes between adherent monolayer and spheroid cultures of MSCs.

WikiPathway	Significance	Genes up in Sph	Genes up in Adh
Glycosaminoglycan metabolism	29.29	7	0
Cell cycle	15.78	62	9
TCF dependent signaling in response to WNT	14.95	3	7
Integrin cell surface interactions	11.76	3	2
Focal adhesion	11.34	26	54
Pyrimidine metabolism	9.82	3	42
EPH-Ephrin signaling	7.36	2	5
VEGFA-VEGFR2 signaling pathway	7.16	28	50
Senescence and autophagy in cancer	6.86	31	14
Transcriptional regulation by RUNX3	6.23	3	4
ESR-mediated signaling	6.13	6	6
Sphingolipid metabolism	6.07	4	1
MAPK6/MAPK4 signaling	5.92	2	5
Extracellular matrix organization	5.51	2	4
Interleukin-1 family signaling	5.45	5	0
Glycerophospholipid biosynthesis	5.29	5	4
Senescent-associated secretory phenotype	4.62	5	1
PI3K-Akt signaling pathway	4.58	43	50
Transcriptional regulation by MECP2	4.47	4	4
JAK/STAT	4.35	16	23
TGF-beta signaling pathway	4.16	14	30
Prostaglandin synthesis and regulation	3.5	13	6
Signaling by EGFR	3.46	4	3
Regulation of actin cytoskeleton	2.71	13	30
Leptin signaling pathway	2.53	12	14
Wnt signaling	2.46	14	20
IL-1 signaling pathway	2.38	13	6
Amino acid metabolism	2.17	7	21
Arachidonic acid metabolism	2.04	3	2

Supplementary Table 3. The top 20 up-regulated and down-regulated genes in MSC spheroids from different passages compared to adherent monolayer MSCs (Fold change).

Gene	Name	Sph P3	Sph P5	Sph P7
CHI3L1	chitinase 3-like 1 (cartilage glycoprotein-39)	1865	1557	873
SLC16A6	solute carrier family 16, member 6	719	491	300
CXCL8	chemokine (C-X-C motif) ligand 8	301	1240	1357
TNFAIP6	tumor necrosis factor, alpha-induced protein 6	247	327	276
PRSS35	protease, serine 35	182	223	211
GDF15	growth differentiation factor 15	172	120	98
BMP2	bone morphogenetic protein 2	172	173	113
SLC16A6	solute carrier family 16, member 6	151	106	66
MMP13	matrix metallopeptidase 13	146	399	413
BMP2	bone morphogenetic protein 2	141	138	80
PDK4	pyruvate dehydrogenase kinase, isozyme 4	130	67	44
CXCL8	chemokine (C-X-C motif) ligand 8	123	371	413
PCSK1	proprotein convertase subtilisin/kexin type 1	104	44	15
AKR1C2	aldo-keto reductase family 1, member C2	99	95	88
FAM20A	family with sequence similarity 20, member A	98	29	5
AKR1C2	aldo-keto reductase family 1, member C2	97	98	91
AKR1C1	aldo-keto reductase family 1, member C1	94	94	87
ABCA1	ATP binding cassette subfamily A member 1	88	48	41
NR4A2	nuclear receptor subfamily 4, group A, member 2	84	83	54
RASD1	RAS, dexamethasone-induced 1	84	76	59
KIF18B	kinesin family member 18B	-90	-97	-107
NCAPG	non-SMC condensin I complex subunit G	-93	-95	-113
CCNB1	cyclin B1	-95	-105	-110
CDK1	cyclin-dependent kinase 1	-97	-167	-162
SPC25	SPC25, NDC80 kinetochore complex component	-100	-90	-112
MYBL1	v-myb avian myeloblastosis viral oncogene homolog-like 1	-106	-109	-92
KRTAP2-3	keratin associated protein 2-3	-115	-81	-45
CCNA2	cyclin A2	-116	-60	-92
SHCBP1	SHC SH2-domain binding protein 1	-121	-128	-113
PBK	PDZ binding kinase	-125	-201	-175
NDC80	NDC80 kinetochore complex component	-140	-162	-149
TOP2A	topoisomerase (DNA) II alpha	-140	-82	-101
HMMR	hyaluronan-mediated motility receptor (RHAMM)	-141	-141	-163
NEK2	NIMA-related kinase 2	-142	-151	-163
DEPDC1	DEP domain containing 1	-149	-134	-157
CEP55	centrosomal protein 55kDa	-150	-259	-249
ASPM	abnormal spindle microtubule assembly	-150	-159	-170
NUF2	NUF2, NDC80 kinetochore complex component	-161	-161	-146
DLGAP5	discs, large (Drosophila) homolog-associated protein 5	-166	-255	-210

ANLN	anillin actin binding protein	-186	-183	-165

Supplementary Figure 2.

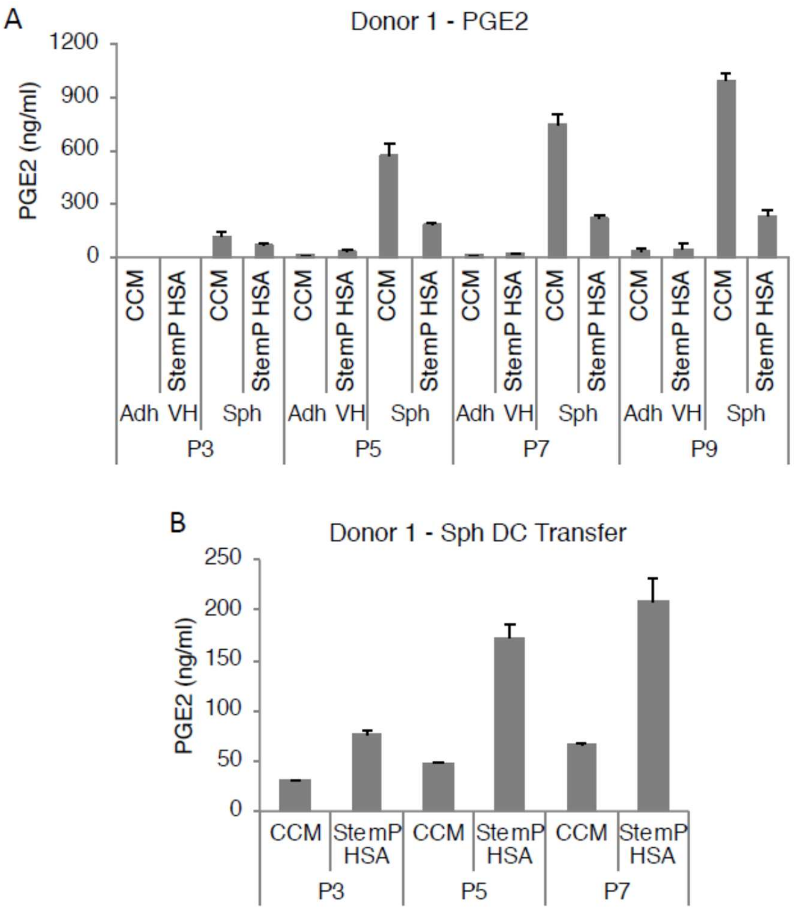

Supplementary Figure 3.

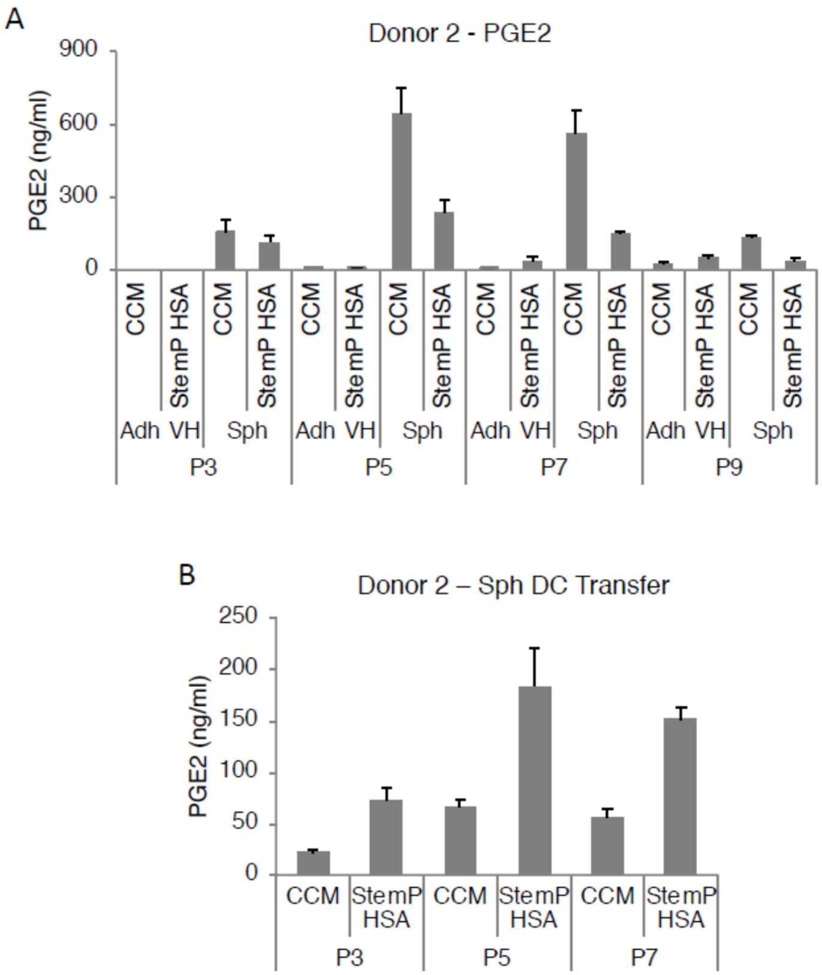

Supplementary Figure 4.

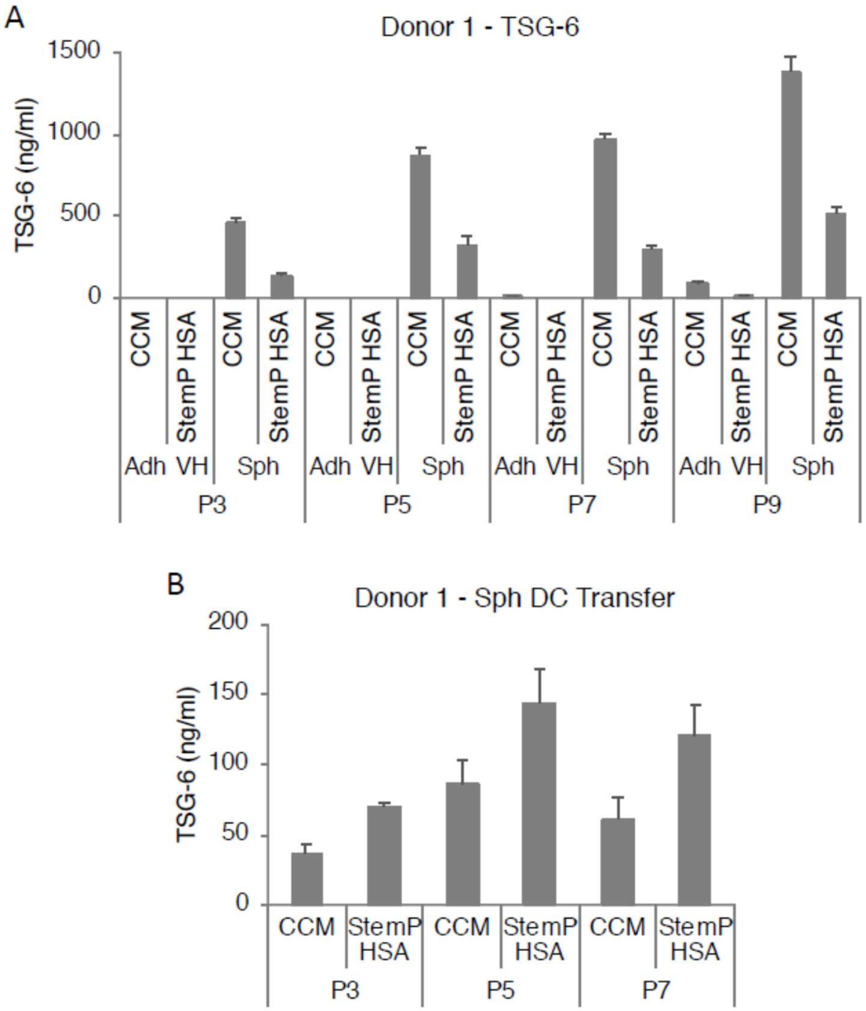

Supplementary Figure 5.

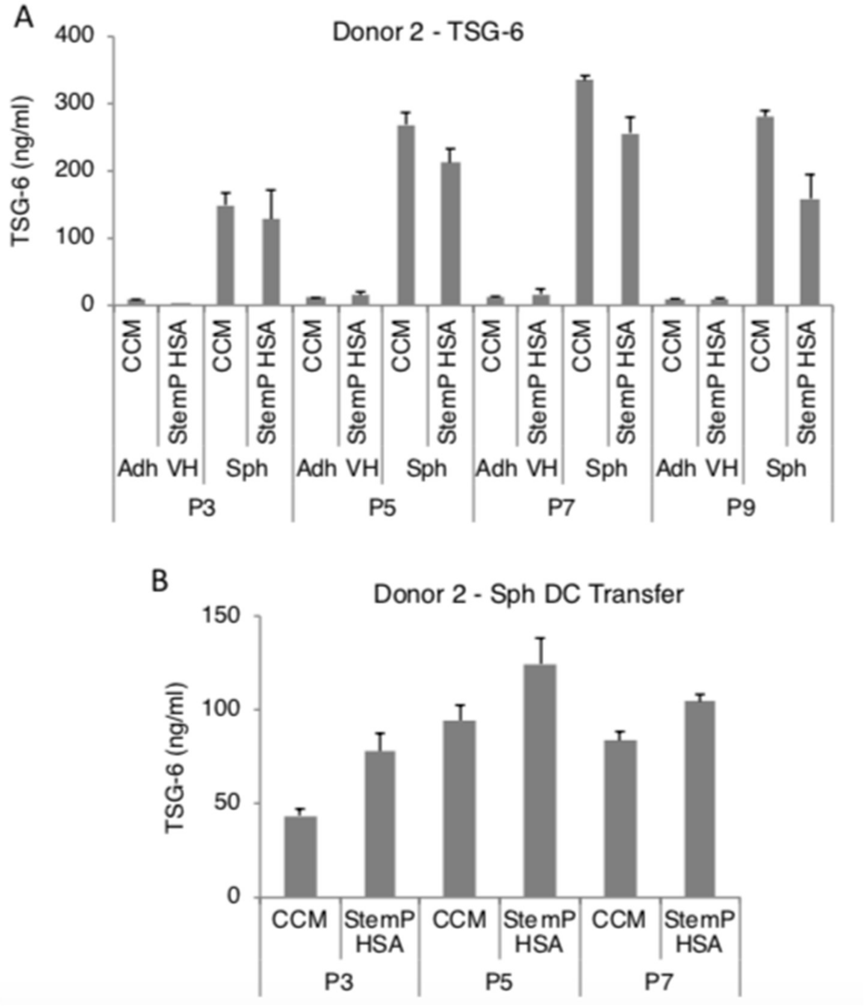

Supplementary Figure 6.

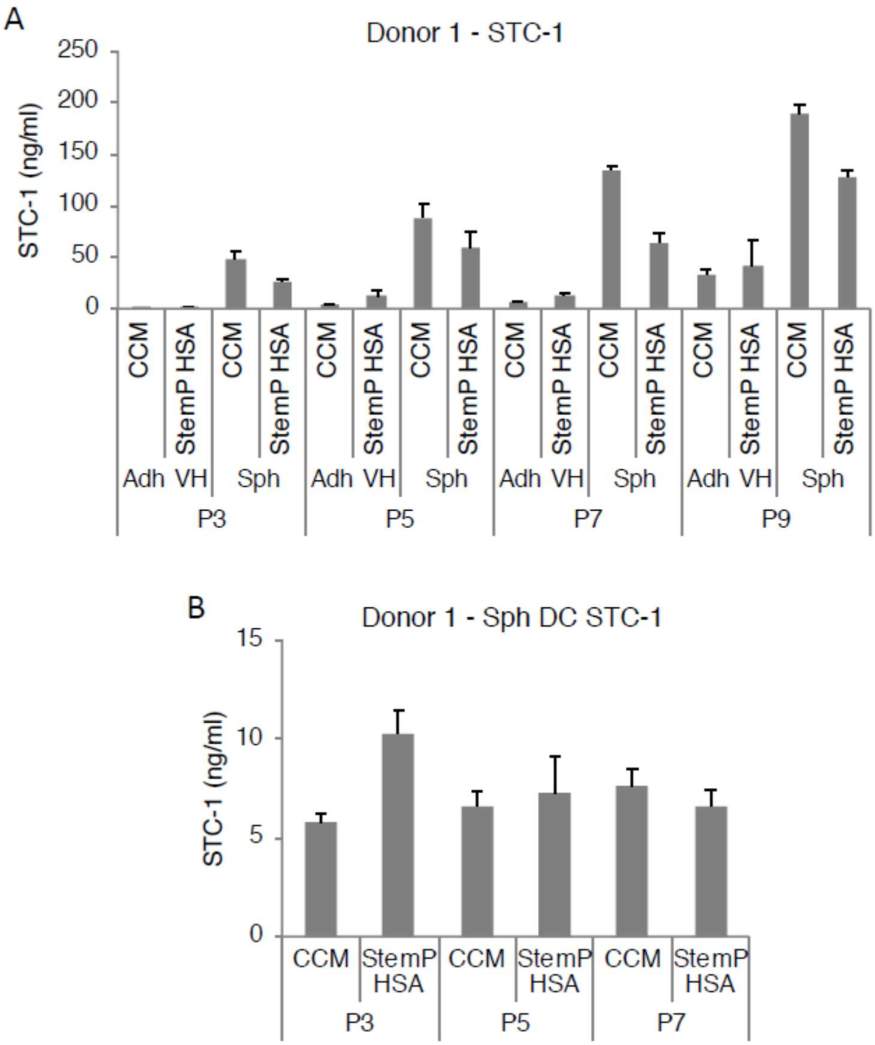

147 Supplementary Figure 7.

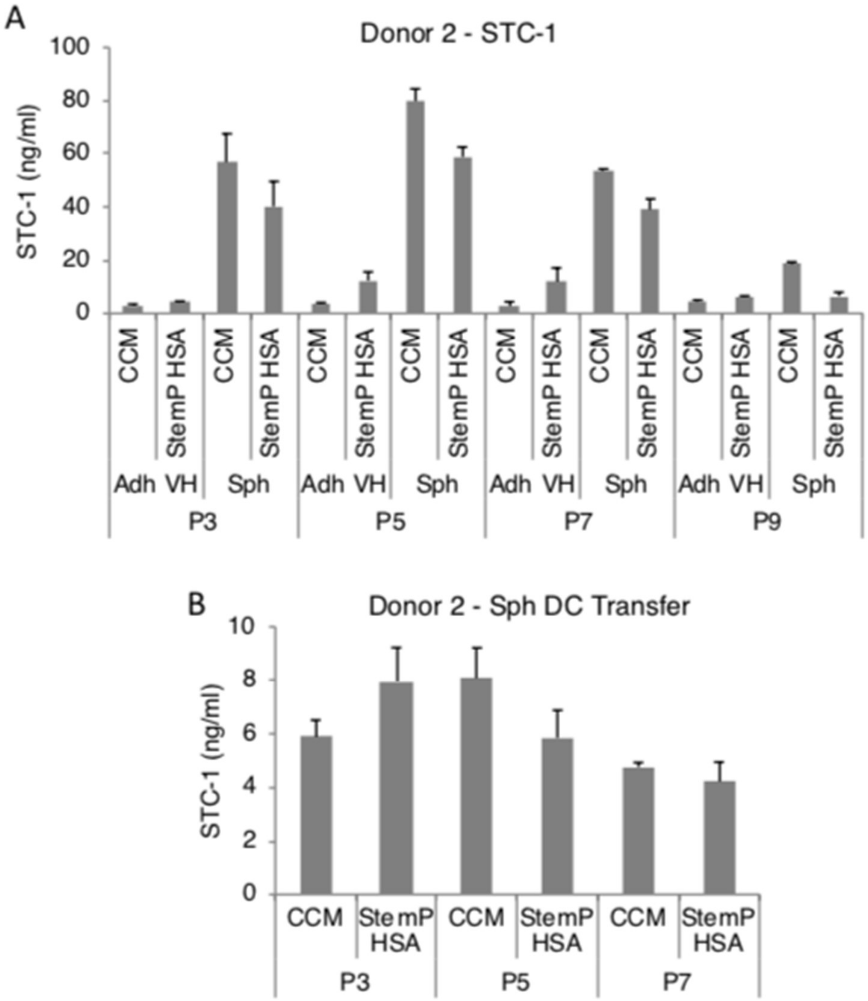

148
